# Documentation of *en route* mortality of summer chum salmon in the Koyukuk River, Alaska and its potential linkage to the heatwave of 2019

**DOI:** 10.1002/ece3.6751

**Published:** 2020-09-11

**Authors:** Peter A. H. Westley

**Affiliations:** ^1^ Department of Fisheries College of Fisheries and Ocean Sciences University of Alaska Fairbanks Fairbanks Alaska USA

## Abstract

This paper documents a mass *en route* mortality event of adult summer chum salmon (*Oncorhynchus keta*) returning to the Koyukuk River, Alaska in the Yukon River basin. In response to reports from local communities, a small team of researchers (including the author) surveyed ca. 275 km of river on July 26 and 27, 2019 and counted 1,364 dead salmon. Although the total magnitude of mortality is unknown, counts from the survey certainly represent only a small fraction of the true number of fish that died. We sampled 73 carcasses to confirm death occurred prematurely prior to complete maturation and spawning, and to quantify sex and length. Visual inspection revealed a substantial fraction exhibited patterns of fungal growth consistent with secondary infections of skin lesions caused by the ubiquitous natural bacterial pathogen *Flavobacterium columnare*. Water temperatures during the survey averaged 17.1°C and the water was approximately 85% saturated with oxygen (ca. 8.5 mg/L), which likely contributed to the stress for upstream migrants. Evidence suggests size‐selective *en route* mortality as female migrants that died were 2% and male migrants 5% shorter than individuals that survived to their spawning grounds on Henshaw Creek. This translates to very strong estimates of natural selection using standardized selection differentials, yet it is unclear whether selection acts on body size directly or indirectly through correlated phenotypic traits such as run timing. The mortality event likely underpins the below average returns of summer chum salmon to the Koyukuk River in 2019, suggesting an impact on spawner abundance. The future consequences of this, or potentially increasingly frequent, *en route* mortality events for population productivity and the extent to which genetic adaptation or adaptive phenotypic plasticity of migration behavior may facilitate persistence of these populations is unknown.

## Graphical abstract

Large numbers of migrating adult summer chum salmon died prematurely en route to spawning grounds on the Koyukuk River in 2019, most likely associated with extreme temperature, low dissolved oxygen, and temperature‐mediated pathogen growth. Death was size selective, favoring larger individuals.

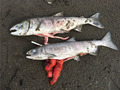

## 1 INTRODUCTION

The frequency and magnitude of mass animal mortality events appear to be on the rise, with abnormal weather conditions and heat stress a leading causal mechanism behind die‐offs in fishes (Fey et al., 2015). Prespawning mortality events in Pacific salmon (genus *Oncorhynchus*) are well documented throughout the ranges of species, but the mechanisms and consequences of these events for populations and ecosystems, including people, are not well known (Bowerman, Keefer, & Caudill, 2016). Prespawning mortality events can occur at various stages of the upstream homeward migration. Upon reaching the spawning grounds, prespawning mortality (PSM) appears linked to spawner density and streamflow, which in turn influences the availability of life‐supporting dissolved oxygen (Sergeant, Bellmore, McConnell, & Moore, 2017). Our understanding of PSM on the spawning grounds is largely limited to years with anomalously large returns (Quinn, Eggers, Clark, & Rich, 2007; Tillotson & Quinn, 2017) or locations where spawner densities are influenced by returns of hatchery‐produced salmon (McConnell, Westley, & McPhee, 2018). While our understanding of PSM on the spawning grounds is in its early phases, most of what we know has been garnered by studies tracking fish *en route* upstream during the freshwater phase of the homeward migration (Hinch et al., 2012).

Substantial work on large watershed complexes such as the Fraser River (British Columbia, Canada), Klamath River (USA), and the Columbia River (USA) basins has revealed that *en route* mortality events are the result of complex interactions between the phenotype of migrants (e.g., run timing and physiology) and their environment, with water temperature and flow being profound variables influencing the biology of migration (Keefer, Peery, & Heinrich, 2008). As a poignant example, early migrating sockeye salmon (*O. nerka*) in the Fraser River tend to die at much higher rates than later returning individuals, which appears to be associated with a mismatch between current warm water conditions and thermal adaptation in terms of aerobic scope to historical migration conditions (Eliason et al., 2011). In contrast, later‐timed migrants of sockeye salmon died at higher rates in the Columbia River during 2000, likely due to delayed migration and increased susceptibility to parasites and pathogens (Keefer et al., 2008). Perhaps the best known example of a temperature‐related disease outbreak is from the Klamath River in September of 2002, where an estimated 34,056 migrating Chinook salmon (*O. tshawytscha*) died as a result of heavy infections of *Ichthyopthirius multifiliis* (Ich) and/or *Flavobacterium columnare* (hereafter, columnaris) (Guillen, 2003). Of course, a perennial challenge is disentangling whether pathogen presence and disease are the proximate agent of mortality or, more commonly in wild fish, a response to other unquantified stressors. Taken as a whole, strong evidence indicates that the combination of high water temperatures, accelerated maturation and senescence, elevated stress, ionoregulatory dysfunction, and disease are the causal agents behind *en route* mortality in migrating salmon (Hinch et al., 2012). Moreover, patterns observed thus far indicate that populations at the southern margins of the ranges may be most susceptible to *en route* mortality given regional warming that increasingly push water temperatures beyond tolerance thresholds. In this paper, I document a large‐scale *en route* mortality event by summer chum salmon (*Oncorhynchus keta*) returning to spawning grounds on the edge of the Arctic in the Yukon River basin, Alaska.

The summer of 2019 will forever be burned in the consciousness of Alaskans. Most apparent was the extreme air temperature anomalies with many regions, including the Yukon, experiencing many days with maximum temperatures in excess of 32°C. Air temperatures in July and August smashed temperature records, ocean temperatures in Alaska were 5 degrees warmer than average, wildfires burned throughout the state choking the air with smoke, droughts plagued rain forests, a lack of sea ice in the Bering Sea, and anomalous biological patterns were detected across the region (Thoman & Walsh, 2019). Beginning on July 20th, the residents living along the Koyukuk River that have long been the stewards and first responders on their lands began reporting a large‐scale die‐off of chum salmon on social media outlets. Hundreds of comments and similar observations streamed in, and on July 26th, a small team was dispatched comprised of a fishery biologist and tribal advocate, the Yukon River summer salmon manager for the State of Alaska, and a salmon evolutionary‐ecologist (the author), traveled to the Koyukuk River drainage with the goals of (a) documenting the the die‐off and assessing its potential magnitude, (b) inferring any apparent abiotic or biotic causes of the event, and (c) quantifying whether mortality was associated with body size or sex by comparing them to observations of fish that survived to the spawning grounds.

## 2 METHODS

### 2.1 Site description

**FIGURE 1:**
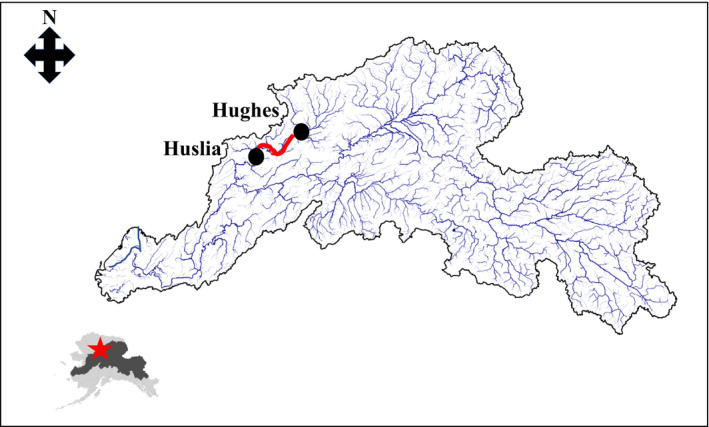
Approximately location (shown as red star on Alaska inset with the Yukon River basin shown as gray polygon) of the 2019 survey that documented *en route* mortality of summer chum salmon. The survey initiated in the village of Hughes and proceeded downstream on the Koyukuk River ca. 275 km (shown as red line) to the village of Huslia

**FIGURE 2:**
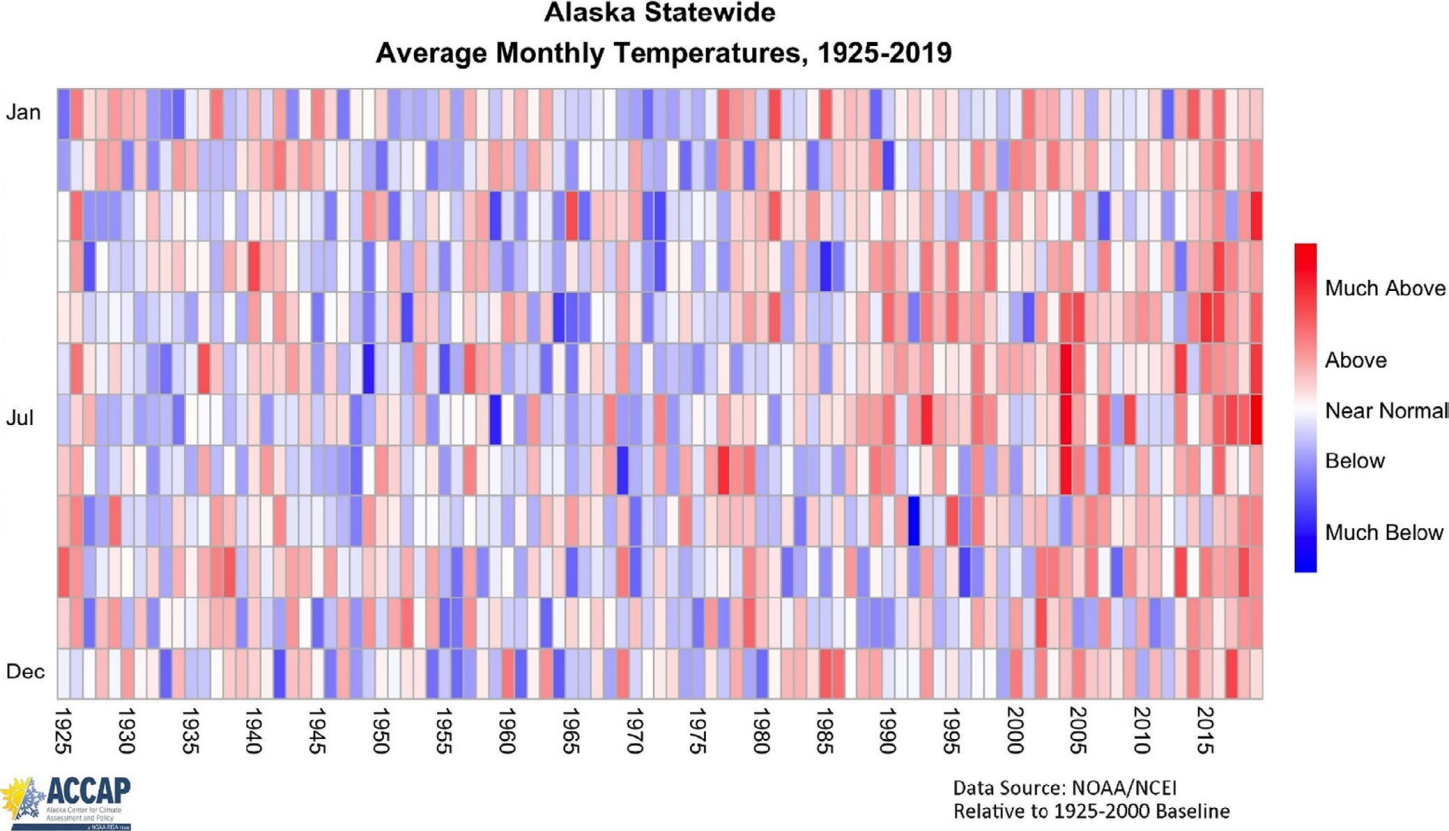
Monthly air temperature anomalies from 1925 to 2019

The Yukon River basin is massive, draining approximately 850,000 km^2^ and flowing more than 3,000 km from its source in Canada to the Bering Sea of Alaska (Figure 1). Made famous in the haunting words of resident Syndey Huntington (Huntington, 1993), the Koyukuk River drains 90,650 km^2^ and flows over 1,000 km before its confluence with the Yukon River where water flows another ca. 775 km to the ocean. On average, the Koyukuk River produces approximately 25% of the summer chum salmon returning to the Yukon River Basin (Larson, Carroll, Conitz, & Borba, 2017). Weirs on the Gisasa River and Henshaw Creek also allow the quantification of spawner numbers and documentation of age, sex, and length of returning adults to the Koyukuk Basin. Examination of size‐selective *en route* mortality (see below) was done only using data from Henshaw Creek as our survey was done upstream of the Gisasa River confluence with the Koyukuk. No systematic monitoring of water temperatures exists on the Koyukuk River, thus our inference that 2019 was an anomaly primarily rests on air temperature records (Figure 2).

### 2.2 Documenting en route mortality and carcass sampling

Our survey was guided by the help of two local knowledge holders, a father and son, whom have lived the entirety of their lives on the Koyukuk River. We surveyed approximately 275 km via a shallow draft jet boat, beginning at the village of Hughes (66.05 N, −154.28 W) and traveling downstream to the community of Huslia (65.69 N, −156.38 W). While traveling, we counted the number of carcasses floating in the river and those that were clearly visible along sandbars and shore banks. On an ad hoc basis and as time allowed, we stopped and surveyed along the river banks where we, (a) counted the number of carcasses along approximately 0.5 km upstream and downstream of the landing site and noted characteristics of carcasses including external signs of scavenging or disease (e.g., fungal growth on fins or gills), (b) recorded the sex and maturation status of dead fish confirmed through internal examination, (c) recorded water temperature and dissolved oxygen (mg/L) using a handheld YSI PRO 2030 model multimeter, and (d) measured the body length of carcasses to the nearest millimeter between the middle of the eye and fork of the tail. Due to decomposition of the tail on 36 individuals, length was measured between the middle of the eye and the hypural flexure and adjusted to mid‐eye fork length using the equation derived from the data (*r* ^2^ = .92):

$${\text{MEF}}_{\text{est}}=1.00*{\text{MEH}}_{\text{obs}}+52.5$$

where MEF_est_ is the estimated middle eye to fork (mm) and MEH_obs_ is the observed middle eye to hypural measurement (mm).

### 2.3 Selection and the role of body size in migration

Binomial logistic regression following Janzen and Stern (1998) was used to investigate the potential role of body size mediating migration success (a proxy of absolute fitness) of upstream migrating individuals. Specifically, the fate of individuals as alive versus dead was coded binomially as a zero or one and used as the response variable in a generalized linear model. Fixed effects of measured body size (at weirs for the alive individuals and from surveys for the dead individuals) and a categorical effect of sex were included in the model with binomial error structure and a logit link function.

To quantify the magnitude of the estimated selection acting on length, I calculated the standardized selection differential following Kendall, Hard, and Quinn (2009), using the equation

$$\left({\bar{X}}_{\mathrm{Alive}}‐{\bar{X}}_{\mathrm{Dead}}\right)/SD_{Alive+Dead}$$

where ${\bar{X}}_{\mathrm{Alive}}$ is the mean (mm) of fish measured at weirs on the spawning grounds and ${\bar{X}}_{\mathrm{Dead}}$ is the mean of (mm) fish to have died *en route*, and $SD_{Alive+Dead}$ is the pooled standard deviation of all length measurements (alive + dead). Standardized selection differentials measure total selection acting on traits through both direct and indirect agents. To quantify uncertainty in selection estimates, I conducted 10,000 randomization simulations through the following procedure. For each simulation, the pooled distribution of all lengths (alive + dead) was sampled with replacement 500 times and used to calculate means for alive and dead individuals that were then used to calculate a standardized selection differential. The observed standardized selection differential calculated from the field was compared to the simulated distribution of differentials to better understand the probability of the observed strength of selection occurring through chance alone. Strength of selection was compared to a recently compiled dataset by Siepielski et al. (2017).

## 3 RESULTS

Consistent with local observations of a significant die‐off event, we encountered the first carcasses floating mid‐river nearly immediately upon leaving Hughes. In the ca. 275 km of survey, we counted 1,364 dead fish.

**FIGURE 3:**
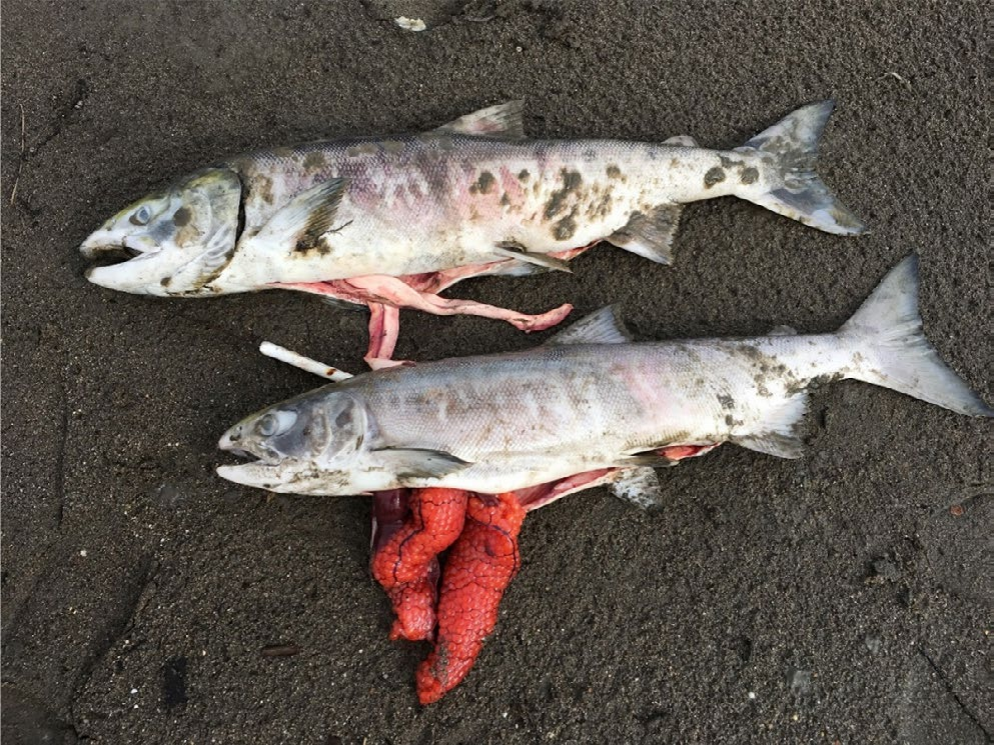
Photograph of male (top) and female (bottom) summer chum salmon surveyed on the Koyukuk River, July 26, 2019. Note the immature status of the testes on the male and ova of the female. Also note the patches of fungus consistent with secondary infection with the bacterial pathogen columnaris *Flavobacterium columnare*. Photograph credit Stephanie Quinn‐Davidson

Sampled 73 carcasses, which varied markedly in condition and state of decomposition. None of the carcasses had obvious signs of trauma or post‐mortem scavenging by animals, a pattern that was also widely noted by local residents. All 73 individual carcasses examined internally for sex had not completed maturation, indicated by firm testes in males and unovulated eggs in females (Figure 3). Approximately 1/3 of the carcasses had signs of potential fungal growth, consistent with secondary infections following the growth of columnaris *(*James Winton, USGS, personal communication).

The water temperature in the mainstem Koyukuk River during the survey averaged 17.1°C (range 16.8–17.4). Dissolved oxygen averaged 8.3 mg/L (Range 7.9–8.6). Given that DO saturation at 17.1°C occurs at 9.65 mg/L, our measurements indicate the water was on average approximately 87% saturated.

**FIGURE 4:**
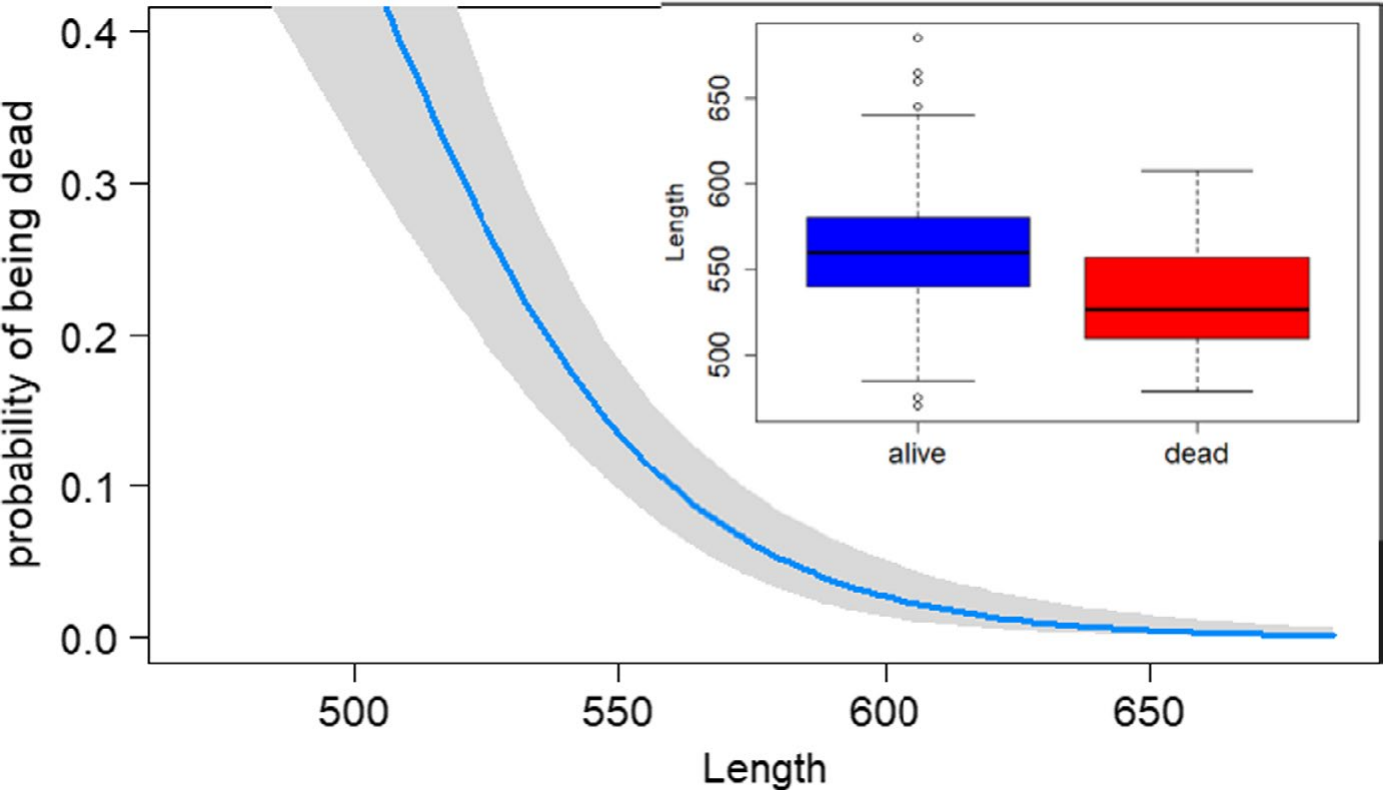
Probability of *en route* mortality declined with size. The blue line is the median estimate from a binomial logistic regression with effects of body length and sex and the gray polygon is the 95% confidence interval of the estimate. Inset boxplots of length of fish that were measured alive at the Henshaw Creek weir (blue) and those surveyed dead *en route* on the Koyukuk River mainstem (red)

Consistent with size‐selective mortality, individuals that died *en route* were ca. 4% smaller on average compared to individuals that survived to the spawning grounds (Table 1). Body size was significantly and inversely associated with probability of death (Figure 4, *p* < .0001) Estimates of standardized selection for males were 0.77, and 0.86 for females. These estimates exceed the 99th percentile of estimates reported by Siepielski et al. (2017) and is thus evidence of very strong selection. However, simulation analysis indicates that our observations of selection could have occurred approximately 25% of the time due to chance alone, which likely reflects the natural wide distribution of body sizes in summer chum salmon.

**TABLE 1:** Sample size (*N*), body length in mm (mean ± *SD*), and date range in which observations were made of summer chum salmon that were measured alive on the spawning grounds of Henshaw Creek or recovered dead during en route migration in the Koyukuk River in 2019

	Alive	Dead
Male	*n* = 276	*n* = 36
Length mean ± *SD*	569 ± 30	545 ± 34
Period of observation	July 3–August 1, 2019	
Female	*n* = 277	*n* = 32
Length mean ± *SD*	532 ± 28	521 ± 25
Period of observation	July 26–27, 2019	

## 4 DISCUSSION

This paper documents substantial *en route* mortality of migrating summer chum salmon in a major tributary of the Yukon River, the Koyukuk River, in Alaska during the summer of 2019. Although the true numbers of fish that died during this event and the proximate mechanisms of the mortality are not known, the existing evidence is consistent with a temperature‐mediated phenomenon. Comparisons of body size between individuals that successfully accomplished their migrations versus those that died *en route* revealed evidence of size‐selective mortality favoring larger body sizes, but whether this reflects direct selection acting on size or indirect selection acting on correlated phenotypic traits is unclear. The ultimate question of how this event will impact future summer Chum salmon production in subsequent years remains to be seen. The lower than average escapement counts observed on the intensely monitored spawning grounds of Henshaw Creek and the Gisasa River suggests that the *en route* mortality event was substantial enough to impact the number of spawners surviving at the population level, which raises conservation concerns should die‐offs increase in their frequency or magnitude..

**FIGURE 5:**
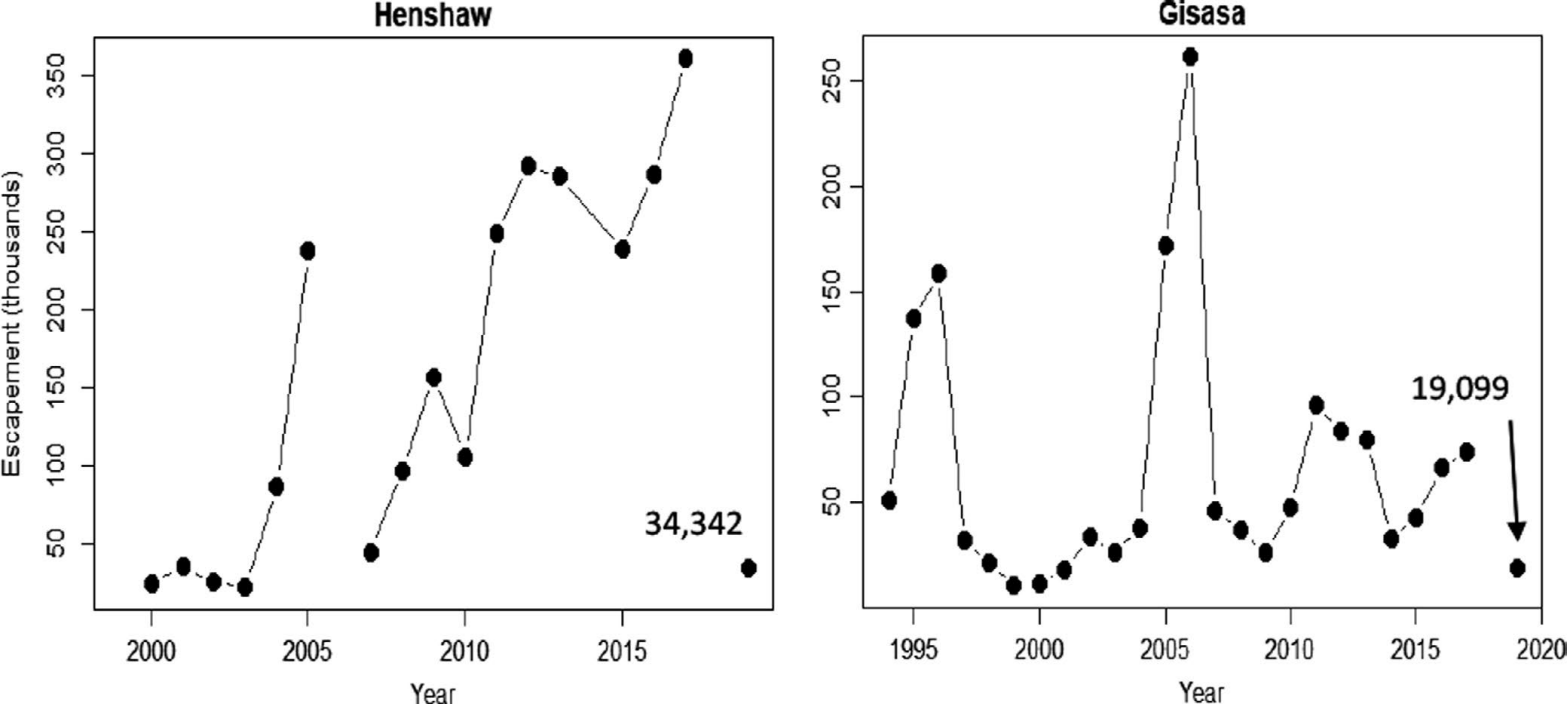
Annual escapement estimates (in thousands) of summer chum salmon for the Henshaw Creek (1999–2019) and Gisasa River (1994–2019). Estimates for 2019 are 34,342 in Henshaw Creek and 19,099 in Gisasa River

Over 1,000 dead fish were counted along a 275 km section of the Koyukuk River during a rapid response survey on July 26–27, 2019. This work would not have been possible without the invaluable observations of local residents and the use of social media to communicate among communities and with scientists and managers. Local residents have always been the first responders to extreme events, and mechanisms to support communities' on‐going stewardship of the river and its salmon are needed more than ever. The magnitude of the die‐off is unknown, but likely substantial given several lines of evidence. First, the survey occurred over a short time period (ca. 1.5 days) and over a relatively small portion of the migratory path. Second, according to local residents and the observed state of carcasses decomposition, it was clear that the survey occurred after the peak of the event, suggesting that counts would have been higher if we had been able to survey the river a few days earlier. Third, carcass counts while traveling were an unknown fraction of the number deposited on shore, which in turn was an unknown fraction of the total number that died throughout the drainage. Given the scarcity of data, it is impossible to estimate what the total fraction of the returning fish succumbed to *en route* mortality. However, observed counts at the two major spawning grounds were below average (Figure 5), which was attributed by managers to the warm temperatures and observed *en route* mortality (Brenner, Larsen, Munro, & Carroll, 2020). Between 1999 and 2018, Henshaw Creek averaged 159,000 spawners, and between 1994 and 2018 Gisasa River averaged 66,736 spawners. The observations of 34,342 and 19,099 spawners were the fourth smallest on record for both Henshaw Creek and the Gisasa River. It is possible that the anomaly between average and observed escapements was larger for the Henshaw Creek population given the greater energetic demands for populations that must travel further upstream to spawning grounds compared to other populations (Crossin et al., 2004) in addition to the difference in accumulated time in stressful migrating conditions (Hinch et al., 2012).

Although the proximate mechanisms of this *en route* mortality event are not known, existing evidence is strongly consistent with temperature‐mediated effects observed in other locations (Hinch et al., 2012). First, hypoxic conditions may have contributed to the stress of migrants given that measurements of dissolved oxygen indicated approximately 87% saturation. It is important to note that migrating individuals require substantially more oxygen than individuals that are not actively migrating (Brett, 1972). That being said, evidence clearly indicates that cardiac failure rather than decreased environmental oxygen is the causative mechanism for death in salmon migrating at warm temperatures (Eliason, Clark, Hinch, & Farrell, 2013). Second, summer chum salmon returning to the Koyukuk River were approximately 8 days later than average (Brenner et al., 2020), which is consistent with the widely observed pattern of delayed migrations in other systems during periods of anomalously warm water. For example, Chinook Salmon in the Klamath River during 2002 entirely ceased migration when the water temperature rose above ca. 20°C, which in turn increased their densities in cool water holding refugia and contributed to the spread of pathogens, particularly Ich and columnaris that ultimately inflicted mass mortality (Strange, 2010, 2013). Third, observed water temperatures during the time of the survey averaged 17.1°C, which is above the optimum temperature for migration and is known to put individuals at risk of increased mortality from pathogens such as columnaris (Holt, Sanders, Zinn, Fryer, & Pilcher, 1975). Approximately, a third of the carcasses were noted to have patches of fungal growth, consistent with earlier infections with columnaris or other bacterial pathogens (Figure 3), but formal pathology was not conducted. Future rapid response surveys should be prepared to take pathology samples to provide diagnostic disease assessments. Importantly, the individual fish we sampled as carcasses on the mainstem Koyukuk River had already traveled over 1,000 km from the mouth of the Yukon River and it is likely that the accumulated time traveling in warm water, combined with delayed migration, increased the susceptibility to pathogens, similar to *en route* mortality events in the Fraser and Columbia rivers.

We detected evidence of size‐selective mortality, where individuals that died *en route* were approximately 4% shorter than individuals that survived to the spawning grounds at the Henshaw Creek weir (Figure 4). This pattern was observed in both males and females and was estimated to be strong selection compared to a global database of standardized selection differentials (Siepielski et al., 2017). However, several important caveats with the analysis are necessary. First, like many analyses of selection this one is plagued by small and unbalanced sample sizes (68 dead vs. 553 alive). Randomization simulations conducted on the combined dataset (alive + dead) revealed that the level of selection observed was likely to occur 1 out of every 4 times due to chance alone. Second, the carcass survey occurred over a much narrower range of dates than the observations at the weir, which may bias results particularly because the size and age of individuals returning to spawning grounds often covary with run timing (Quinn, McGinnity, & Reed, 2015). Indeed, the size of returning individuals to the Henshaw Weir declined significantly throughout the run (*p* < .001, *r* ^2^ = 0.06). However, analyses using a date‐truncated dataset from the Henshaw weir revealed similar patterns of size selection with large fish being favored to return, and thus the pattern was consistent while including the potential effect of run timing. Third, it is not clear whether size selectivity is the result of direct selection acting on body size or is the result of both direct and indirect selection acting on correlated phenotypic traits such as physiological tolerances and others that comprise a migratory phenotype. The selection analysis here assumes only direct selection, which is likely not true. Future work to survey carcasses throughout the run and to gather additional phenotypic measurements would be needed to formally calculate selection gradients that allow quantification of direct and indirect influences on selection.

Studies that report size‐selective upstream *en route* mortality associated with stressful water temperatures are rare. Although substantial work has been done to quantify the magnitude and causes of die‐offs in locales such as the Fraser and Columbia rivers, quantification of size‐selective mortality is lacking. This is presumably due to the logistical challenges of carcass collection in large rivers as well as the uncertainty of assigning individuals to populations of origins. The use of radio telemetry to track the fate of individuals to their spawning grounds circumvents some of these challenges, and English et al. (2005) reported no significant difference in size between fish that survived to the spawning grounds versus those that were detected to have died *en route*, for either the summer run or late run of sockeye salmon in the Fraser River. In contrast to body size, evidence suggests energetic condition‐dependent mortality in the Fraser River with the prediction that larger body size and condition should be advantageous (Rand et al., 2006). Notwithstanding the caveats discussed previously, the evidence from the Koyukuk River suggests that bigger tends to be better with regards to migratory survival. Future work to associate body size and energetic condition would be insightful as well as additional years of data on size‐dependent *en route* mortality to provide more context for what was observed in 2019.

Will the *en route* mortality that occurred in 2019 have a lasting consequence on the populations in the Koyukuk River and how might populations adapt to warming? While this event gives cause for concern given it appears to have resulted in substantially below average spawner escapements, there are also reasons for optimism. Pacific salmon are remarkably productive and resilient in the face of disturbance (Hilborn, Quinn, Schindler, & Rogers, 2003), and there is little evidence that small spawner numbers can result in positive density‐dependent effects, also known as Allee Effects or depensation (Cunningham, Ruggerone, & Quinn, 2013). The variable age of adult migrants contributing to any one return year (e.g., fish that spent 2, 3, or 4 winters at sea) provides a natural buffer that will likely dampen (or mask) the true effect of the die‐off in future years (Schindler et al., 2010). In contrast, species such as pink salmon (*O. gorbuscha*) that all mature at two years of age might be particularly vulnerable to mortality events. Consistent with observations from other systems in the southern part of the Pacific salmon range, it seems the most obvious response to this and potential future events are shifts in run timing by adults to avoid migrations during stressful periods (Quinn & Adams, 1996). Indeed, run timing and migratory behavior, which reflect both plastic and genetic components, are predicted to shift earlier in many systems (Crozier, Scheuerell, & Zabel, 2011; Reed, Schindler, Hague, et al., 2011). Of course, the extent to which run timing may shift is a function of the strength of selection and heritability in addition to plasticity (Carlson & Seamons, 2008; Reed, Schindler, & Waples, 2011). It is important to recall, however, that run timing is in part also shaped by the selection acting on other parts of the life history, such as the timing of spawning given the water temperature during incubation (Brannon, Powell, Quinn, & Talbot, 2004). This presents potential conflicts, as selection may favor earlier return timing on the adult life history to avoid stressful water temperatures, whereas warming should select for *later* timing given the relationship with temperature, embryo development, and match‐mismatch dynamics. This dynamic selection tug‐of‐war is worthy of additional exploration using eco‐evolutionary modeling.

While the short‐term or long‐term consequences of 2019 remain to be seen and understood, it is increasingly clear that regional warming throughout Alaska and the Arctic is likely to make these events more frequent and of greater severity. Preparations need to be made to avoid, to the extent possible, surprises like this that are on the horizon. This stark reality underscores the need to protect habitats that serve as the template for life history variation that buffers against disturbance (Moore, Yeakel, Peard, Lough, & Beere, 2014), ensure migrating individuals have natural options to seek thermal refugia in cooler waters during stressful periods (Armstrong, Ward, Schindler, & Lisi, 2016), and support local human communities to continue being the first responders and stewards of salmon, without which all of us would be impoverished.

## CONFLICT OF INTEREST

None declared.

## AUTHOR CONTRIBUTIONS

**Peter A. H. Westley:** Conceptualization (equal); data curation (equal); formal analysis (equal); investigation (equal); writing‐original draft (equal).

## Data Availability

Data are archived and freely available on the Knowledge Network for Biocomplexity: https://doi.org/10.5063/F1BG2MB2.
